# miRNAs and Novel Food Compounds Related to the Browning Process

**DOI:** 10.3390/ijms20235998

**Published:** 2019-11-28

**Authors:** Silvia Lorente-Cebrián, Katya Herrera, Fermín I. Milagro, Juana Sánchez, Ana Laura de la Garza, Heriberto Castro

**Affiliations:** 1 Department of Nutrition, Food Science and Physiology/Centre for Nutrition Research, Faculty of Pharmacy and Nutrition, University of Navarra, 31008 Pamplona, Spain; slorente@unav.es (S.L.-C.);; 2 Navarra Institute for Health Research, Navarra Institute for Health Research, 31008 Pamplona, Spain; 3 Centro de Investigación en Nutrición y Salud Pública, Facultad de Salud Pública y Nutrición, Universidad Autonoma de Nuevo Leon, 64460 Monterrey, Mexico; katya.herreravld@uanl.edu.mx (K.H.);; 4 Nutrition Unit, Center for Research and Development in Health Sciences, Universidad Autonoma de Nuevo Leon, 64460 Monterrey, Mexico; 5 CIBERobn, Fisiopatología de la Obesidad y la Nutrición, Carlos III Health Institute, 28029 Madrid, Spain; 6 Laboratory of Molecular Biology, Nutrition and Biotechnology (Nutrigenomics and Obesity), University of the Balearic Islands, 07122 Palma, Spain; joana.sanchez@uib.es; 7 Instituto de Investigación Sanitaria Illes Balears, 07020 Palma, Spain

**Keywords:** miRNAs, browning, BAT, WAT, food compounds

## Abstract

Obesity prevalence is rapidly increasing worldwide. With the discovery of brown adipose tissue (BAT) in adult humans, BAT activation has emerged as a potential strategy for increasing energy expenditure. Recently, the presence of a third type of fat, referred to as beige or brite (brown in white), has been recognized to be present in certain kinds of white adipose tissue (WAT) depots. It has been suggested that WAT can undergo the process of browning in response to stimuli that induce and enhance the expression of thermogenesis: a metabolic feature typically associated with BAT. MicroRNAs (miRNAs) are small transcriptional regulators that control gene expression in a variety of tissues, including WAT and BAT. Likewise, it was shown that several food compounds could influence miRNAs associated with browning, thus, potentially contributing to the management of excessive adipose tissue accumulation (obesity) through specific nutritional and dietetic approaches. Therefore, this has created significant excitement towards the development of a promising dietary strategy to promote browning/beiging in WAT to potentially contribute to combat the growing epidemic of obesity. For this reason, we summarize the current knowledge about miRNAs and food compounds that could be applied in promoting adipose browning, as well as the cellular mechanisms involved.

## 1. Brown and Beige Adipose Tissue in Energy Balance

BAT is a type of adipose tissue characterized by multilocular lipid droplets, the abundance of mitochondria, and a high rate of fatty acid oxidation and glucose uptake. It secretes batokines and exhibits endocrine, paracrine, and autocrine actions. In addition, it plays an important role in energy expenditure and non-shivering thermogenesis and is considered to have potential implications for the treatment of obesity [1].

**Figure 1:**
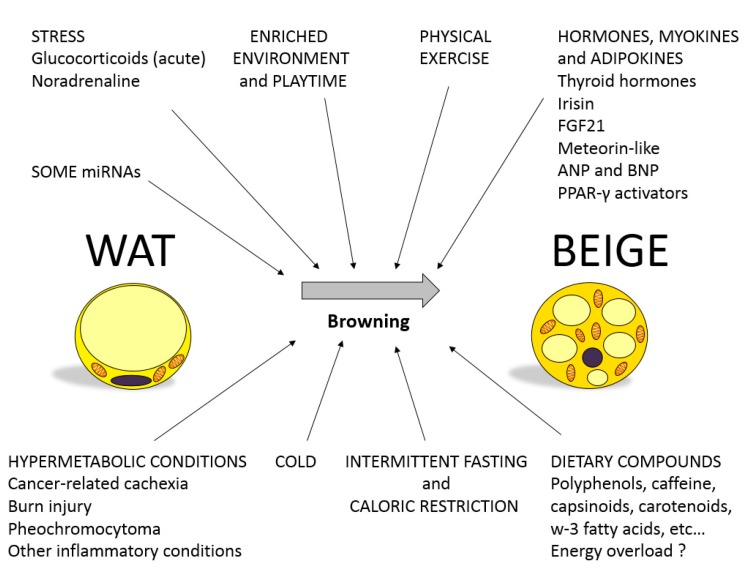
Most relevant environmental, behavioral, and physiological factors that regulate white adipose tissue (WAT) browning. miRNAs, microRNAs; FGF21, Fibroblast growth factor 21; ANP, atrial natriuretic peptide; BNP, brain-type natriuretic peptide; PPAR-γ, Peroxisome proliferator-activated receptor gamma.

Moreover, brown adipocyte-like cells, beige or brite adipocytes, are a specialized cell type derived from white-adipocyte precursor cells that display thermogenic capacity [2]. Functionally, brite adipocytes have the ability to efficiently oxidize glucose and lipids [3] to produce heat under certain stimuli [4] (see Figure 1 and below). Further, providing their thermogenic capacity, stimulation of these brite adipocytes (widely known as beiging or browning) has eventually been considered as an interesting strategy against excessive energy accumulation observed in obesity [5]. From a morphological point of view, brite adipocytes appear within white adipose tissue depots (mainly in the subcutaneous depot), have multilocular smaller lipid droplets (as compared to white adipocytes), display medium mitochondrial density, and express (inducible) uncoupling protein-1 (*Ucp-1*) [2].

The myogenic factor 5 (Myf5) give rise to brown adipocytes and also a subset of white adipocytes. Individual brown and white adipocytes contain a mixture of adipocyte progenitor cells derived from Myf5^+^ and Myf5^neg^ lineages, which varies depending on the depot location [6]. Thus, adipose tissue development towards a more white/brite phenotype depends on a complex interaction/combination between environmental and transcriptional factors, including epigenetic mechanisms such as miRNAs [7] and dietary bioactive compounds such as polyphenols [8].

As explained in Figure 1, multiple factors have been related to browning activation in WAT [4], a negative energy balance being a common trait to many of them. Among the most relevant ones, this includes physical exercise, cold, stress hormones (noradrenaline and glucocorticoids), caloric restriction, and intermittent fasting. In this context, it has been speculated whether, in some cases, WAT browning is a negative adverse side effect given that it has been observed in severe-wasting situations such as burn-injured individuals, cancer-associated cachexia, or pheochromocytoma [9]. However, in general, the browning process is considered a promising strategy to revert the positive energy balance typical of obesity, and many studies have been devoted in the last years to study hormones, miRNAs, and physiological stimuli (i.e., physical activity and social stimulation) that are able to activate browning, including also dietary compounds that are involved in trans-differentiation from white to beige adipocytes.

## 2. MicroRNAs as Regulators of Gene Function and Metabolism

MiRNAs are non-coding ribonucleotid acid (RNA) molecules that regulate gene expression. To clarify, MiRNAs are short, approximately 20 to 22 nucleotides in length, double stranded RNAs that usually down-regulate gene expression at the post-transcriptional level [10]. For the last decade, many hundreds of miRNAs have been identified with highly conservative sequences between species. Moreover, this high conservation degree shows important roles of miRNAs in the regulation of tissue development, phenotype, and cell physiology [11], including adipose tissue [12,13,14].

In most cases, miRNAs repress gene transcription through specific and direct interaction of miRNA and mRNA. This miRNA–mRNA interaction is based on base pair complementarity, and usually, miRNAs interact with target mRNAs at their 3′UTR region [10,11]. However, other non-canonical regions for miRNA interaction have also been described, such as the coding region or the 5′UTR [15]. The canonical miRNA mechanism of action involves a perfect base pair complementarity within the “seed” sequence, framed within base pairs 2–8 of the mature miRNA strand, with the target mRNAs sequence. Additionally, some mismatches could be present within the non-seed sequence of the miRNA strand for the regulation of gene transcription. Indeed, one single miRNA can regulate many mRNAs, and at the same time, one mRNA could be regulated by several miRNAs and, thus, affect different cell functions [10,11,16,17,18]. Even so, this suggests that miRNAs constitute a wide and open system for transcriptional regulation of gene expression in mammals. The biological impact of gene transcription regulation through miRNAs demonstrates the plasticity of this system to finely tune gene expression under environmental changes.

## 3. Involvement of miRNAs in the Regulation of Browning: Role of Nutritional Factors

**Figure 2:**
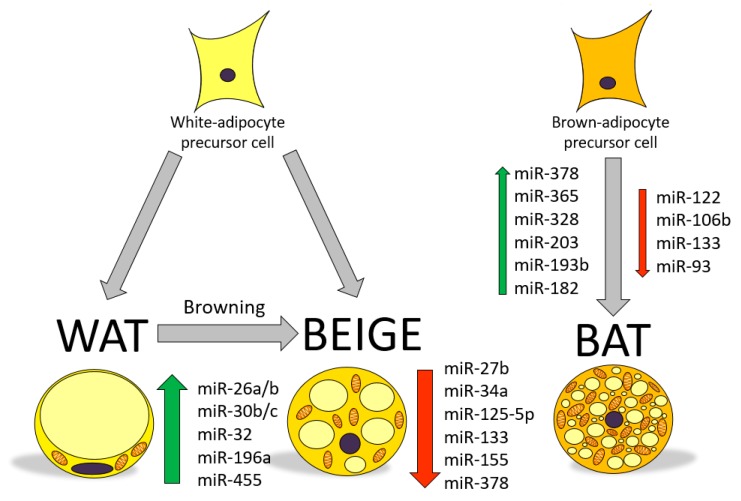
MicroRNAs involved in WAT browning and beige and brown adipocyte regulation. ↑, upregulated; ↓, downregulated.

In recent years, many studies have demonstrated the involvement of miRNAs in the regulation of adipose tissue function and adipose tissue formation (adipogenesis) [12,13,19]. Of particular relevance, miRNAs regulating brite adipocyte development and function have been a topic of interest for obesity research [20]. Therefore, identification of specific miRNAs regulating brite/brown adipocyte function could be envisaged as a potential therapeutic tool to improve the excess of WAT associated to obesity and to stimulate energy dissipation as heat [21]. The role of miRNAs in the modulation of browning relies on their ability to tiny regulate gene transcription, while some miRNAs have been found to be positive regulators of brite/brown adipocytes (activators), others have shown to be potent inhibitors of adipocyte browning [22,23]. Figure 2 displays some of the miRNAs found to regulate brite/brown function that are briefly described in this review.

Importantly, several studies have also shown that nutritional and dietary factors could influence miRNAs associated with browning, thus, potentially contributing to the treatment of excessive adipose tissue accumulation (obesity) through specific nutritional and dietetic approaches. Table 1 and Table 2 illustrate the most important nutritional factors that regulate browning as well as brown and brite adipocyte-related miRNAs, respectively, as observed in human samples and cells. Table 3 shows additional information about these miRNAs in animal and murine cell models.

## 4. Food Compounds Related to the Browning Process

Evidence has shown that some food compounds could be promising candidates to target different molecular mechanisms and dietary strategies in the management of obesity, and, even more, some of them have the potential ability to promote the browning process in WAT. For instance, some of the most studied compounds are spices, green tea, polyunsaturated fatty acids (PUFA´s), carotenoids, and citrus fruits, among others.

### 4.1. Spices

#### 4.1.1. Chili Peppers

Capsaicin (trans-8-methyl-*N*-vanillyl-6-nonenamide) is a bioactive component of chili peppers [77,78], and is also the major pungent component of red-hot chili peppers. Additionally, chili peppers contain non-pungent capsinoids analogs, which include: capsiate, dihydrocapsiate, and nordihydrocapsiate [78,79]. It is widely accepted that capsaicin and capsinoids activate BAT and increase thermogenesis, as well as energy expenditure in both rodents and humans [79,80,81,82]. In addition, there is increasing evidence regarding the browning effect of capsaicin and capsinoids.

Baboota et al. [83] reported that male Swiss albino mice fed a high-fat diet (HFD) receiving 2 mg/kg body weight of capsaicin (orally administered on alternate days; capsaicin was dissolved in 0.9% saline with 3% ethanol and 10% Tween 80) for 12 weeks had an induction of the browning phenotype in subcutaneous WAT, but not in the visceral WAT, and showed increased on thermogenesis and mitochondrial biogenesis genes expression in BAT.

Baboota et al. [84] also showed that 3T3-L1 adipocytes treated with 1 µM of capsaicin increased the expression of *Ucp-1*, *Pgc-1α*, *Prmd16*, *Dio2*, *Pparα*, and *Foxc2*, not only suggesting that capsaicin induced a brown-like phenotype but that this effect was via the transient receptor potential vanilloid subfamily 1 (TRPV1)-dependent mechanism.

Baskaran et al. [85] confirmed in mice that the activation of TRPV1 channels by capsaicin triggers browning of WAT. Adult male TRPV1-/- mice were fed an HFD with added capsaicin (0.01%) from week 6 to week 32. Dietary capsaicin increased the expression of *Ucp-1*, *Pgc-1α*, *Sirt-1*, and *Prdm16* in WAT wild type mice. Hence, capsaicin had no effect on the expression of these genes in TRPV1-/- mice. Thus, TRPV1 protein may have a central role in capsaicin-mediated browning of WAT.

Mosqueda et al. [86] described that adult male Wistar rats that were fed a Western diet for eight weeks and treated daily with capsaicin (4 mg/kg/day) induced in retroperitoneal WAT the emergence of multilocular brown-like adipocytes that were positive for UCP-1 and CIDEA. In addition, the increased expression of *Prdm16* was observed in the inguinal WAT of these animals.

Fan et al. [87] reported that mature 3T3-L1 adipocytes were tested with a combination of capsaicin (25 µM) and capsiate (25 µM) for 48 h increased the expression of *Tbx1*, *Tmem26*, *Cd40*, *Ucp-1*, and *Prdm16*, in 3T3-L1 adipocytes and also increased the protein levels of UCP-1, PGC-1α, and PRMD16. The induction of browning in white adipocytes by a combination of capsaicin and capsiate could be mediated via activation of the peroxisome proliferator-activated receptor gamma/beta3 adrenoreceptors (PPARγ/β3-AR) signaling pathway.

Ohyama et al. [88] described a synergistic anti-obesity effect by a combination of capsinoids and mild cold exposure promoting beige adipocyte biogenesis. Adult male C57BL/6J mice were fed a high-fat diet with 0.3% (*w*/*w*) of capsinoids (62.7% of capsiate, 32.2% of dihydrocapsiate, and 5.5% nordihydrocapsiate) for eight weeks. Due to a mild cold exposure, 17% of these animals had increased expression of *Ucp-1*, *Pgc-1α*, and *Cidea*, in the inguinal WAT when compared with vehicle-treated mice and mice kept under room temperature. Cells treated with nonivamide (the less pungent capsaicin-analog), decreased the adipogenesis process through an increase in the expression of the miRNA mmu-let7d-5p, which has been associated with decreased PPARg levels [89].

#### 4.1.2. Turmeric

Curcumin is a polyphenol isolated from the rhizomes of the plant *Curcuma longa*, commonly known as turmeric, a member of the ginger family (*Zingiberaceae*). It is widely used as a spice in Asian cuisine [90,91]. Curcumin is practically insoluble in water [92] and has poor absorption into the gastrointestinal tract in rats, displaying a low oral bioavailability [93]. In vitro and animal studies support the hypothesis that curcumin may promote thermogenesis and/or the browning process. For instance, Wang et al. [94] reported that mice treated with curcumin (50 or 100 mg/kg/day) for 50 days displayed a lower body weight gain and lower fat mass without any effect of food intake, and also showed better tolerance to cold exposure. Particularly, in these animals, curcumin induced browning of the WAT but in a depot-dependent manner, since increased mitochondrial biogenesis and increased expression of *Ucp-1* and other brown-fat specific genes were found in inguinal but no in epididymal WAT (eWAT). In addition, these animals presented higher circulating levels of norepinephrine and increased expression of the β3-AR in the inguinal WAT, suggesting that the browning of the inguinal WAT (iWAT) is mediated by the norepinephrine-β3-AR pathway. Moreover, Nishikawa et al. [95] also reported that the oral administration of curcumin formulations with higher bioavailability than native curcumin increased browning in iWAT tissue of mice. Song et al. [96] reported that male C57BL/6J mice fed with a high-fat diet plus 1% of curcumin presented higher thermogenic capacity in response to a cold challenge, already evident after two weeks of treatment. In addition, a long-term dietary intervention (17 weeks) did not affect iWAT browning but increased *Ucp-1*, *Ppara*, and *Pmrd16* expression in the BAT. About miRNAs, there are no reports concerning the browning process; however, it has been seen that curcumin markedly increased the content of miRNA-34a in SGC-7901 cells, inhibited proliferation, migration, and invasion. As described above, miRNA-34a has been studied in human WAT and expressed negatively with waist circumference [49,97].

In vitro studies have shown that curcumin also induces browning and mitochondrial biogenesis in 3T3-L1 cell line and primary inguinal white adipocytes [98,99]. Curcumin significantly increased several brown fat markers in both adipocytes in a dose-dependent manner (doses ranged from 1 to 20 µM). In fact, this effect seems to be mediated via the AMP-activated protein kinase (AMPK) pathway [99]. In addition, a proteome analysis of primary inguinal white adipocytes treated with 20 µM curcumin for six to eight days revealed that curcumin increased protein levels related to fatty acid oxidation, lipolysis, and browning-specific markers [98].

#### 4.1.3. Thyme

Thymol (2-Isopropyl-5-methylphenol) is monoterpene phenol, and the major constituent of essential oils isolated from plants belonging to the *Lamiaceae* family such as Thymus (*Thymus vulgaris*) [100,101]. *Thymus vulgaris* is a native plant of Mediterranean regions traditionally used as a culinary herb and also in traditional medicine [101]. Not to mention, it is also used in the food industry for its flavoring and preservative properties [100]. Therefore, it has not been deeply studied regarding its effect on body weight control, thermogenesis, or browning effect, and, in addition, there is no evidence related to any miRNA. However, Choi et al. [102] described that thymol might promote fat browning and lipid metabolism in 3T3-L1 adipocytes. Meanwhile, adipocytes treated with 20 µM of thymol increased mRNA and protein levels of PGC-1α, PRMD16, and UCP-1. In addition, there is a synergic increase of *Fgf21*, *Pgc-1α*, *Prdm16*, *Tmem26*, and *Ucp-1* (brow fat-specific genes) in the presence of 20 µM of thymol together with 50 nM triiodothyronine and 1 µM rosiglitazone (browning cocktail). Overall, the authors suggest that thymol might promote the browning of white adipocytes via activation of the β3-AR pathway.

#### 4.1.4. Cinnamon

Cinnamon is produced from the bark of trees from the genus *Cinnamomum* and belongs to the family *Lauraceae* [103]. The major components are cinnamaldehyde and cinnamic acid [104]. Regarding the bioactive compounds of cinnamon, recent studies showed biological effects of cinnamaldehyde, such as anti-inflammatory, antioxidant, and anti-obesity properties [105]. Also, several hydroxycinnamic acid derivatives, such as cinnamic acid, ameliorated the obesity induced by an HFD in rats [106]. Furthermore, trans-cinnamic acid (tCA), as an analog of cinnamon, demonstrated beneficial properties on body weight and blood lipids in obese rats [104]. In this context, regarding the molecular mechanisms of cinnamon and its bioactive compounds, several in vitro studies have been developed on murine adipocytes and found that cinnamaldehyde (40 µM) prevents adipocyte differentiation through the downregulation of *Ppar-γ* and Ccat-enhancer-binding protein alpha (C/EBPα) [107]. Regarding the browning process, *Prdm16* interacts directly with *Ppar-γ* and plays an important role in the thermogenesis process [108]. Thus, in an in vitro study, tCA induced browning in 3T3-L1 cells in a concentration of 200 µM upregulated protein expression of PGC-1α, PRDM16, and UCP-1 and upregulated genes involved in the browning process and beige-fat-specific like *Pgc1-*α, *Prdm16*, and *Ucp-1* [104]. Similarly, in an in vivo study, cinnamaldehyde supplementation (10 mg/kg BW) downregulated adipogenic genes and promoted lipolysis and brown adipose tissue activity. These results led to decreased body weight gain and fat accumulation after 14 weeks of treatment [107]. Likewise, another study showed that cinnamaldehyde supplementation (40 mg/kg/day) decreased body weight by inducing the browning of WAT in HFD-fed mice [108]. However, although the effects of cinnamon and its bioactive compounds on the browning process have been identified, to date, the relationship with miRNAs has not been described. Not only was it not directly in an obesity model, but a recently published article also showed that cinnamaldehyde regulates inflammation in macrophages through the suppression of miR-21 and miR-155 [109]. Not to mention, the study focused on inflammatory bowel disease and ulcerative colitis. As a result, a relationship has been found with the suppression of miR-155 in the process of browning [110].

#### 4.1.5. Garlic

Bioactive compounds from garlic (*Allium sativum* L.) have been studied for their possible anti-obesity effect in in vitro models, animal models, and clinical trials [107]. The most abundant biocompounds present in garlic are allicin, alliin, diallyl sulphide, diallyl disulphide, diallyl trisulfide, ajoene, and S-allyl-cysteine [111]. In this context, some of these compounds have been shown to exert effects on lipid accumulation, such as ajoene, in concentrations from 25 µM to 200 µM in 3T3-L1 cells [112]. Furthermore, other in vitro studies have been developed to investigate the effects of garlic-derived organo-sulfur compounds. Thus, the results report a decrease in intracellular triglycerides after diallyl trisulphide in adipocytes [107]. Additionally, the authors highlighted the possible molecular mechanisms to decrease gene expression related to adipogenesis, as well as the increase in genes related to fatty acid oxidation, such as *Ucp-1* [107]. Also, in relation to garlic biocompounds and its anti-obesity properties, it has been identified that thiacremonone enhanced the mRNA level of uncoupling protein-2 (*Ucp-2*) in 3T·-L1 cells [113]. Similar results have been found in in vivo studies, suggesting an anti-obesity effect of garlic supplementation. In this regard, M. Kim and Kim demonstrated a potential anti-obesity effect of garlic (2% or 4%) in obese mice, downregulating adipogenic genes. Likewise, in another study with obese mice treated with garlic powder (2% or 5%) for seven weeks, lower body weight, WAT, and adipogenic gene expression was found in WAT and increased *Ucps* mRNA levels and AMPK activity in BAT [114]. All these studies have demonstrated the effect of garlic and its biocompounds on the browning process in different in vitro and in vivo models. However, although the modulation of miRNAs by garlic has been identified, its effects have not been related, so far, with the browning process. More studies are needed to highlight the effect of garlic and its biocompounds on miRNAs and BAT.

#### 4.1.6. Onion

Quercetin is one of the most abundant flavonoids present in food (onions, apples, tea, and red wine) [115] and is the aglycone form of other glycosidic flavonoids. In this context, as an example, onion (*Allium cepa* L.) is a natural source rich in phenolic compounds, fructans, and organosulfur compounds. Phenolic compounds from onion, especially flavonols such as quercetin, are secondary metabolites that have been widely studied for the anti-obesity effects [116]. Possible anti-obesity mechanisms of quercetin could involve AMPK and mitogen-activated protein kinase (MAPK) signaling pathways. AMPK is a key regulator of energy balance inducing mitochondrial biogenesis and, therefore, promoting BAT. In this context, Lee et al. [117] showed increased expression of genes related with brown adipocyte development, such as *Prmd16*, *Ucp-1*, and *Pgc1-α* in retroperitoneal white adipose tissue (rWAT) of mice fed with HFD supplemented with 0.5% onion peel extract (OPE) for eight weeks. In addition, the same authors demonstrated the conversion of white to brown adipocytes in 3T3-L1 cells cultivated with 50 µM of quercetin during the differentiation process (6 days) [117]. Also, another in vivo study found lower triglycerides levels in mice fed HFD supplemented with quercetin (0.1% *w*/*w*) for 12 weeks. This result may be mediated by the increased expression of *Ucp-1* in sWAT [118]. Although the role of miRNAs as a potential mechanism involved in the regulation of the browning process has not been specially described, some miRNAs could be implicated in the inflammatory response [119]. In a recent study, the effect of quercetin on miR-155 expression in RAW264.7 macrophages stimulated with lipopolysaccharide (LPS) was evaluated. Thus, they demonstrated that treatment with quercetin (10 µM) down-regulated the expression of miR-155 in LPS-stimulated macrophages [73]. Another study showed that quercetin and isorhamnetin (a quercetin metabolite) improved the inflammatory status induced by LPS in murine macrophages, and suggested miR-155 as a regulatory inflammatory mediator [120]. Although, in these studies, miR-155 was related to the macrophage response to various types of inflammatory markers. Likewise, other studies described that the inhibition of miR-155 could enhance brown adipocyte differentiation through the browning process [110,121]. On the other hand, a recent study identified an upregulated miR-369-3p expression level in dendritic cells treated with quercetin. The foregoing led to the down-regulation of *C/ebp-β*, which resulted in a lower production of inflammatory cytokines [122].

### 4.2. Other Herbal and Food Compounds

#### 4.2.1. Magnolia Officinalis

Magnolol (5,5′-diallyl-2,2′-dihydroxybiphenyl) is a hydroxylated biphenyl active compound isolated from *Magnolia officinalis* [123,124]. In fact, a range of biological activities has been attributed to magnolol, such as antiinflammation, antioxidation, antiangiogenesis, and anticancer activities [123,124]. In addition, it has been described that long-term supplementation of magnolol (17 mg/kg BW per day for 16 weeks) ameliorates body fat accumulation, insulin resistance, and adipose inflammation in HFD fed male C57BL/6J mice [125]. Moreover, regarding the thermogenic or browning effect in the adipocytes, Parray et al. [126] described that 3T3-L1 adipocytes treated with 5–20 µM of magnolol during differentiation displayed a dose-dependent increase in the protein level UCP-1, PGC-1α, PRMD16, and PPARγ. The addition of 20 µM of magnolol in the presence of a 50 nM triiodothyronine with 1 µM rosiglitazone from day six to eight of differentiation in 3T3-L1 cells increased the expression of specific browning markers such as *Ucp-1*, *Pgc-1α*, *Prmd16*, *Cd137*, *Tbx1*, and *Cidea*. In sum, these results suggest that magnolol might promote the browning of 3T3-L1 adipocytes. Also, magnolol increases UCP-1, PGC-1α, and PRDM16 in HIB1B brown adipocytes.

Honokiol ((3′,5-di-(2-propenyl)-1,1′-biphenyl-2,2′-diol) is a phenylpropanoid molecule found in several species belonging to the genus *Magnolia* [127,128]. Firstly, it presents a structural homology with magnolol [129], and magnolol is a structural isomer of honokiol [124]. Secondly, among the different effects attributed to honokiol, it has been suggested that it may promote neuroprotection [127,129] and may exert an anticarcinogenic effect [130]. Furthermore, there is scarce literature on the effect of honokiol on body weight control or its effect on thermogenesis, at least in vivo. Also, in vitro studies on 3T3-L1 adipocytes pointed out an induction of brown adipocyte-like phenotype in white adipocytes by honokiol. In addition, these cells presented a dose-dependent (1–20 µM) increase in the protein levels of PGC-1α, PRDM16, and UCP-1. Moreover, 3T3-L1 adipocytes grown on a media containing 20 µM of honokiol from day six to eight of differentiation presented increased mRNA levels of *Cidea*, *Cox8*, *Fgf21*, *Pgc-1α*, and *Ucp1* (genes characteristics of brown adipocytes) and increased protein levels of C/EBPβ, PPARγ, and peroxisome proliferator-activated receptor delta (PPARδ) (key regulatory proteins for browning). In fact, the authors suggest that the induction browning in these cells could be mediated through the activation of extracellular signal-regulated kinase (ERK).

Likewise, honokiol also increased the levels of PGC-1α, PPARγ, PRDM16, and UCP-1 in HIB1B cells (at 20 µM) (when these cells are fully differentiated, they express *Ucp* RNA like BAT in vivo; thus, these are good cell models for studying the regulation of *Ucp* gene expression in BAT and metabolism [131,132]. Nowadays, it is known that the magnolol induces miR-200c expression in breast cancer cells [133], with regard to this, miR-200c is also related to the browning process in humans [121].

#### 4.2.2. Berberine

Berberine is an isoquinoline alkaloid derived from the Chinese medicinal plant *Coptis chinensis* (Chinese goldthread) [134]. It has been found that berberine inhibits fat accumulation adipocytes in vitro and in diet-induced obese mice and also can induce weight loss in rodents and human subjects [135,136,137,138,139]. Henceforth, recent emerging evidence suggests that berberine increases energy expenditure, thermogenesis, and browning.

Zhang et al. [140] reported that obese db/db mice treated for four weeks with berberine (i.p, administration of 5 mg/kg/day) had higher rectal temperature and presented improved cold tolerance than control animals. In fact, berberine administration also induced the expression of *Pgc-1α*, *Ucp-1*, and *Cidea*, among others, in BAT of obese db/db mice. Also, berberine administration induces browning of iWAT but not in eWAT. In iWAT, obese db/db mice treated with berberine presented increased expression of *Pgc-1α*, *Ucp-1*, and *Cidea*. These animals also had increased mitochondrial biogenesis and a BAT-like phenotype of inguinal adipocytes.

Lin et al. [141] showed that HFD-fed C57BL/6J mice treated with berberine at 100 mg/kg/day in 0.9% saline by oral gavage had reduced obesity and were protected against the development of hyperglycemia, hyperlipidemia, and insulin resistance. In addition, berberine treatment increased oxygen consumption and the expression of *Ucp-1* and tyrosine hydroxylase (rate-limiting enzyme in the heat production process) [142]. The 3T3-L1 cells treated with 5 µM berberine for 48 h had increased expression of CITED1, BMP7, PRDM16, and UCP-1. This effect may be mediated through transcription-coupled post-transcriptional regulation. In another study, a treatment with berberine revealed a decrease in miRNA-92a, which led to an increase in RNA-binding motif protein 4a (RBM4a) expression, promoting the beige adipogenesis in 3T3-L1 [141].

Wu et al. [143] reported that in 10 overweight individuals with NAFLD that received berberine for one month (0.5 g, 30 min before eating, three times a day), there was an increase in the mass and activity of BAT. In addition, HFD-induced obese mice that received berberine for six weeks (i.p, administration of 1.5 mg/kg/day) displayed enhanced BAT thermogenesis and reduced body weight gain. Therefore, these animals presented higher rectal temperature and increased expression of *Ucp-1*, *Prdm16*, *Pgc-1α*, *Ppargc1b*, and *Elovl3*, and higher protein levels of UCP-1 and PRDM16 in BAT compared to their controls. However, berberine administration did not induce the typical browning phenotype in iWAT or eWAT.

#### 4.2.3. *Panax ginseng*

*Panax ginseng* is one of the most used traditional Chinese herbs and has an approximately 2500-year medicinal history in Eastern Asia, and is a slow-growing perennial plant that belongs to the genus *Panax* in the *Araliaceae* family [144]. The majority of *P. ginseng* contains protopanaxadiols and protopanaxatriols as dammarane type triterpene saponins [145]. Among them, ginsenoside Rb1 is the main protopanaxadiol saponin, and ginsenoside Re, Rg1, and notoginsenoside R1 are the main representative protopanaxatriol components [146]. The importance of ginseng lies in its multiple pharmacological functions, such as anticancer activity, as well as antioxidant and aging inhibitory effects [77]. Additionally, fat accumulation in obesity involves two cellular mechanisms: WAT hypertrophy and hyperplasia [147]; likewise, there are several reports showing that ginseng can reduce adipocyte size and fat storage in mice and rats fed with HFD [148]. Therefore, an effect of Rg1 has been demonstrated to suppress the accumulation of triglycerides (TG) in 3T3-L1 cells and the obesity model induced by the HFD of zebrafish [149].

AMPK, a critical regulator of energy metabolism, has been shown to play a key role in adipose tissue metabolism [150]. In fact, AMPKα1 activation has been found to be involved in the regulation of browning in iWAT of mice [151]. As a result, it has been shown that Rg1 induces BAT-like differentiation of adipocytes and thermogenic activation, which is a process that also involves the upregulation of fatty acid oxidation through the activation of AMPK. Consequently, lipid droplets take a form typical of brown adipocytes and *Ucp-1* expression is induced, and mitochondrial activity increases [152]. In this sense, a study revealed that Rb2 activates BAT and induces browning of WAT, which increases energy expenditure and thermogenesis and consequently ameliorates obesity and metabolic disorders, suggesting Rb2 to be a promising beneficial compound treating obesity [153]. In diet-induced obese mice supplemented with Rb2 BAT activation and WAT browning, as shown by reducing lipid droplets, increasing *Ucp-1* staining, and activating brown gene programs [154]. Thereupon, miR-27b is identified to regulate adipogenesis by targeting Ppar-γ2, and in this sense, has shown that expression levels for both of miR-27b and its primary transcript, pri-mir-27b, were found to decrease upon ginsenoside Rb1 treatment in 3T3-L1, which in turn promotes Ppar-γ expression and adipogenesis [155].

#### 4.2.4. Mentha

The commercially important species *Mentha piperita* and *M. arvensis* are cultivated for the essential oil used in food, confection, cosmetics, alcoholic beverage, tobacco, and pharmaceutical industries [156]. The essential oil of members of the mint (*Lamiaceae*) family is dominated by monoterpenes. In fact, menthol is one of the major constituents of essential oil of both species. Despite the similarity in the menthol biosynthetic pathway of these two species, the compositional variations in the essential oil cannot be ignored, as *M. arvensis* essential oil constitutes the major trading essential oil for menthol in the world. Likewise, menthol is a chemical cooling agent naturally produced from mint oils or prepared synthetically [157]. Consequently, a cold sensation in mammals is detected by several cold-sensors; among them, the most preeminent one is the cold and menthol receptor, canonical transient receptor potential cation channel subfamily M member 8 (TRPM8) [158]. Some findings highlight the role of menthol-induced TRPM8 activation in *Ucp-1* dependent thermogenesis and energy expenditure [159]. Activation of TRPM8 receptors in the gut and skin by oral and topical administration of menthol leads to an increase in serum glucagon levels, thus activating several downstream catabolic processes like the browning of WAT and activation of energy expenditure markers in WAT and BAT [160].

Some studies also demonstrated the induction of the browning process through TRPM8 activation on human WAT, highlighting the importance of the presence of TRPM8 in adipose tissue [161,162]. However, to date, no studies have been found that directly relate this species to miRNA and the browning process. Nevertheless, an in vitro study showed that L-menthol administration at bioavailable doses significantly increased browning/brite and the energy expenditure phenotype and enhanced mitochondrial activity-related gene expression [163]. In addition, an in vivo experiment has found that dietary menthol enhanced WAT browning and improved glucose metabolism in HFD-induced obesity mice, which suggests that TRPM8 might be involved in WAT browning by increasing the expression levels of genes related to thermogenesis and energy metabolism [164].

#### 4.2.5. Chrysin

Chrysin (5,7-dihydroxyflavone) is a natural flavonoid found in honey, propolis, mushrooms, and other plant species [165,166]. Also, it has been reported that chrysin exhibits an antioxidant and anti-inflammatory effect, a protective effect against diabetes, and also possesses neuroprotective activity [165,166,167]. In fact, its role in the activation of thermogenesis or browning effect is less studied. Choi et al. [168] reported that 3T3-L1 adipocytes treated with 50 µM of chrysin in the presence of a 50 nM triiodothyronine and 1 µM rosiglitazone (a “browning cocktail”) from day six to eight of differentiation presented increased expression of proteins/genes related to lipolysis and fatty acid oxidation, and decreased the expression of proteins related to lipogenesis. Moreover, chrysin increased the expression of brown fat markers in these cells via a mechanism involving AMPK and PGC-1α. It is important to note that, like the curcumin, chrysin too has been related to the miRNA-34a.

#### 4.2.6. Soy

Isoflavones are a subgroup of flavonoids present in soybeans and other legumes [169]. Genistein is an isoflavone widely studied in different cells and animal models. Some of the biological properties of genistein highlight its anti-obesity effect [170]. In this context, Lephart et al. [171] demonstrated significantly upregulated *Ucp-1* mRNA expression levels in BAT of Long-Evans rat supplemented with soy isoflavone mixture. In a recent in vitro study, genistein programmed adipocytes to be converted into beige adipocytes. In the first place, the treatment consisted of adding genistein at concentrations of 10, 50, or 100 µM during the differentiation period. Furthermore, the molecular mechanisms involved could be a reduction in mRNA levels of genes related to lipogenesis and increased expression of genes that regulate the development of brown adipocytes such as *Cebpβ* and *Pgc-1α* [172]. On the other hand, a recent study evaluated the effect of genistein in an in vivo model in which mice were fed an HFD supplemented with genistein (0.25 g/kg diet) for eight weeks. To point out, the results showed less weight gain, sWAT weight, and free fatty acid levels in mice supplemented with genistein compared to the HFD group. Regarding the mechanisms, the authors emphasize the correlation between the differentially expressed genes in the hypothalamus and browning markers induced by genistein supplementation [173]. 

Daidzein is another isoflavone whose effect on body weight and lipid metabolism has been studied in WAT and BAT. In this context, rats were supplemented with daidzein (5 mg/kg and 50 mg/kg) for 10 weeks. The results showed expression changes in genes involved in adipogenesis and fatty acid oxidation. Furthermore, daidzein treatment increased UCP-1 protein expression in BAT, activating the thermogenesis [174]. Regarding miRNAs modulated by genistein, to date, there is no specific evidence on miRNAs that regulate the browning process and BAT. Nevertheless, genistein was associated with decreasing miR-155 and consequently improved inflammation in an in vitro model with human umbilical vein endothelial cells (HUVECs) [175]. In addition, another study reported the anticancer effects of genistein by modulating miR-155 in breast cancer cells [176]. However, additional studies are needed to analyze the effect of genistein on miR-155 in relation to BAT.

#### 4.2.7. Green Tea Polyphenols

Tea polyphenols have been reported to have many health benefits, such as antioxidant and anti-inflammatory activities [177]. The predominant constituents of green tea, accounting for up to 35% of the dry weight, are the polyphenols, which include flavonols, flavones, and flavan-3-ols commonly known as catechins [178]; these are present also in fruits, such as apple, grapes and berries, beans, cocoa and red wine [179]. In fact, catechins have received much attention due to their beneficial effects on health [180]. The most abundant green tea catechin is epigallocatechin gallate (EGCG), which accounts for about 50% to 70% of green tea catechins [82]. Mice fed 0.5% polyphenolic green tea extracts rich in catechins and EGCG for eight weeks showed whitening of the BAT and induced the browning process in the WAT [181]. Meanwhile, rats supplemented with green tea extract (155 mg/kg/day for eight weeks) showed higher expression of both *Ucp-1* and *Ppar-γ* [182]. In essence, the intake of oolong, red, and black tea has been found to reduce adiposity and foster browning of mWAT in mice, and these effects were accompanied by increased AMPK phosphorylation [183]. In fact, most studies have found greater activation of regulatory genes in the browning effect. A study on mice evaluated the influence of a single oral administration of theaflavins on energy metabolism and found an increase in *Ucp-1* and *Pgc-1α* mRNA levels in BAT. These results indicate that theaflavin rich fraction significantly enhances systemic energy expenditure, as evidenced by an increase in the expression of metabolic genes [184]. On the other hand, 3T3-L1 preadipocyte cell showed significantly reduced intracellular lipid accumulation by targeting miR-27a and miR-27b, as well as Ppar-γ [185]. In recent years, an increasing number of clinical trials have confirmed the beneficial effects of green tea on obesity; however, the optimal dose has not yet been established. However, a clinical trial has shown that the intake of catechin (615 mg) and caffeine (77 mg) 2 times/day for five weeks increases the cold-induced thermogenesis effect [186]. Overall, most of the studies aimed to assess the impact of green tea catechins on fat oxidation rather than thermogenesis; therefore, more studies are necessary to evaluate their involvement in the browning process in humans.

### 4.3. PUFA’s

#### 4.3.1. Conjugated Linoleic Acid

Conjugated linoleic acids (CLAs) are molecules mostly found in dairy products and ruminant meats [187]. CLA is a group of PUFA derived from linoleic acid (LA, C18:2 n-6), with a conjugated double bond in different cis- and trans-arrangements [188]. Additionally, twenty-eight isomers of CLA have been identified, with the predominant isomers being cis-9, trans-11-CLA (c9, t11-CLA), which represents up to 90% of total CLA in food, and trans-10, cis-12-CLA (t10,c12-CLA), present in a smaller quantity [189]. Some studies have suggested that CLA leads to weight loss by reducing the size and altering the evolution of adipocytes [190]. In fact, proposed anti-obesity mechanisms of 10, 12 CLA isomer include decreased lipogenesis and increased lipolysis [191]. Moreover, *Cidea* has been associated with thermoregulation [192], lipid droplet dynamics, and lipid metabolism [193]. A study in human adipocytes treated with CLA found an upregulation of *Cidea* and *Ucp-1* genes, setting the role of *Cidea* in the transcriptional regulation of thermogenesis [194]. Also, it has been described that CLA induces the browning of WAT, increasing *Ucp-1* and *Cidea* gene expression in both and iWAT, with higher *Ucp-1* mRNA and protein detected in iWAT from 10,12 CLA-supplemented mice [195]. Male mice fed an HFD diet for five weeks and then switched to an LFD with or without 0.1% 10,12 CLA decreased WAT weight and increased mRNA levels of genes related to thermogenesis and fatty acid oxidation in eWAT and iWAT, including *Ucp-1* and *Ppar*-α [196]. Nevertheless, in vitro studies indicate that direct treatment of pre-adipocytes with trans-10, cis-12 CLA increased markers of inflammation and suppressed markers of beiging or browning. A study in human adipocytes showed that 30–50 µM trans-10, cis-12 CLA consistently decreased the mRNA, protein, and activity levels of PPARγ, an adipogenic transcription factor that induces *Ucp-1* expression [197]. Furthermore, in brown adipocytes isolated from hamsters, mRNA levels of *Ucp-1* were increased 260% by 71.4 µM cis-9, trans-11 CLA, but were suppressed 80% by 71.4 µM trans-10, cis-12 CLA compared with the controls [198]. Most studies have been developed in in vivo models indicating positive regulation of browning markers; however, in vitro experiments indicate that the effect of 10,12 CLA is still unclear. Regarding miRNA, CLA mediates its pro-resolving effects in part via regulation of the proinflammatory miR-155, which has been associated with the WAT browning effect [199].

#### 4.3.2. Eicosapentaenoic Acid

Fish oil, rich in omega-3 (n-3) PUFAs such as eicosapentaenoic acid (C20:5n-3, EPA), is anti-inflammatory primarily by reducing the production of proinflammatory cytokines [200]. Furthermore, it has been suggested that EPA is able to enhance energy dissipation in subcutaneous adipose tissue to stimulate oxidative metabolism and reduces fatty acid release by facilitating fatty acid storage in mice [201]. In addition, it has been shown that EPA promoting mitochondrial biogenesis and beige-like markers in human subcutaneous adipocytes from overweight subjects [202]. For this reason, there is evidence that EPA may induce adipocytes to acquire a beige phenotype and may activate brown thermogenesis. In addition, fish oil increased thermogenic markers in the BAT, such as β3-AR and *Ucp-1* [203,204]. C57BL/6 male mice fed with EPA for five weeks improved the expression of genes related to the browning of WAT, and BAT thermogenic markers such as *Ppar-α*, *Ppar-γ*, and fibronectin and fibronectin type III domain-containing 5 (FNDC5) [203]. Zhao et al. [201] reported that EPA significantly increased the expression of thermogenic genes like *Ucp1-3*, *Cidea*, and *Vegfα*. In addition, carnitine palmitoyltransferase 1B (*Cpt1b*) mediates the transport of fatty acids into the mitochondrial matrix for β-oxidation. It has been reported that EPA promotes brite adipogenesis and improves the parameters of mitochondrial function, such as increased expression of *Cpt1b* [205]. On the other hand, miRNAs play critical functions in BAT differentiation and maintenance. The deletion of adipose-specific dicer promotes white-like phenotypes of brown precursor cells [206]. In this context, Kim et al. [76] confirmed the association between miR-30b/378 and brown thermogenesis in C57BL/6 mice fed with fish oil.

#### 4.3.3. Docosahexaenoic Acid

Docosahexaenoic acid (DHA) is a long-chain, highly unsaturated omega-3 (n-3) fatty acid. It has a structure that gives it unique physical and functional properties, as well as 22 carbons in its acyl chain, which includes six double bonds. DHA is found in fairly high amounts in seafood and products derived from seafood. Examples of fatty fish are mackerel, salmon, trout, herring, tuna, and sardines [207]. In fact, DHA is of particular interest, given their potential effects on adipose tissues and metabolism [208]. Also, it has shown a better anti-inflammatory effect than EPA in adipose tissue, which may be related to a more significant role of DHA in promoting *Ucp-1* expression [209]. Additionally, recent studies showed that BAT activation or WAT browning of WAT by n-3 PUFAs were independent of *Ucp-1* [210]. It was shown that refeeding rats a diet enriched in PUFAs, but not in saturated fatty acids, following two weeks of food restriction increased BAT mass and expression of *Ucp-1* [211]. Nonetheless, the thermogenic stimuli via the intervention with DHA and their ability to induce *Ucp-1* in WAT are still controversial [212].

### 4.4. Carotenoids

#### 4.4.1. β-Carotene

Carotenoids are a diverse group of compounds responsible for most of the yellow, orange, and red colors in fruits and vegetables [213]. Among the six most abundant carotenoids in plasma, α-carotene, β-cryptoxanthin, lutein, zeaxanthin, lycopene, and β-carotene [214]. Carotenoids are notably stored in adipose tissue [215]. What is more, carotenoids and carotenoid conversion products impact gene expression and cell function through multiple mechanisms. The most important carotenoid is pro-vitamin A β-carotene, which is metabolized by various enzymes to generate several cleavage products, including retinoic acid (RA) [216]. The effects of carotenoids and retinoids on adipose tissue mostly rely on their ability to bind to specific nuclear receptors, namely RA receptors (RARs) and retinoid X receptors (RXRs), in order to modulate gene expression in the adipocyte [217]. However, it has been suggested that carotene within the BAT could be metabolized to a significant degree to retinoic acid and, thus, become a *Ucp-1* activator [218]. Studies have also reported the effect of carotenoid-related compounds in increasing energy expenditure and thermogenesis in both WAT and BAT; nonetheless, the interaction with miRNA has not been analyzed. The participation of β-carotene in the modulation of *Ucp-1* expression was established by in vitro studies [219]. Furthermore, the treatment with RA in murine BAT cells induces an overexpression of thermogenic and catabolic proteins [220].

#### 4.4.2. Fucoxanthin

Fucoxanthin is a natural pigment that can be extracted from brown seaweeds and is rich in xanthophylls (a subset of carotenoids) [221]. Dietary fucoxanthin is mostly absorbed as fucoxanthinol, a hydrolyzed metabolite in the small intestine, and enters systemic circulation through lymph [222]. Although, it is demonstrated that fucoxanthin is involved in a variety of physiological processes in the body and have many beneficial properties, including the antioxidant and anti-inflammatory and neuroprotective effect [223]. Indeed, fucoxanthin an anti-obesity effect in both in vitro and in vivo in mice and human models [224]. In 3T3-L1 adipocytes, fucoxanthin reduced lipid accumulation accompanied by a decrease in *Ppar-γ* expression [225]. However, the intake of an HFD supplemented with 0.2% fucoxanthin in C57BL/6 mice increased *Ucp-1* expression in both WAT and BAT [221,226]. Maeda et al. [227] indicated that fucoxanthin upregulates the expression of *Ucp-1* in WAT, which may contribute to reducing WAT weight. Overall, obese mice fed with fucoxanthin showed a decrease in WAT weight as well as a significant upregulation in the expression of UCP-1 mRNA and protein in WAT, resulting in an increase in energy expenditure in the form of heat and fatty acid oxidation in WAT [227]. These studies suggest that fucoxanthin may have promising anti-obesity effects and deserve more research focus especially related to miRNAs and primarily in human subjects, as the results are still confusing. For example, Rebello et al. [228] did not find changes in the expression of genes related to WAT browning, fat oxidation, and glucose transport in vitro in primary human adipocytes treated with fucoxanthin.

### 4.5. Citrus Fruits

**Figure 3:**
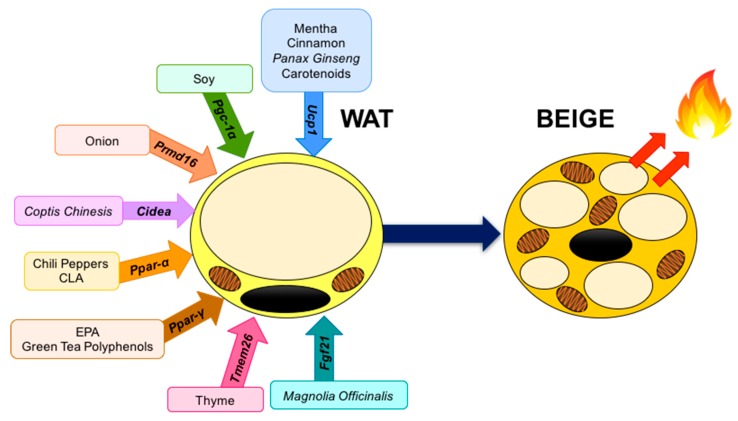
Food compounds and activated genes involved in WAT browning. *Fgf21*, Fibroblast growth factor 21; *Tmem26*, Transmembrane protein 26; *Pparγ*, Peroxisome proliferator-activated receptor gamma; *Pparα*, Peroxisome proliferator-activated receptor α; *Cidea*, Cell death inducing DFFA like effector A; *Prmd16*, PR-domain containing 16; *Pgc-1α*, Peroxisome proliferator-activated γ receptor co-activator 1 alpha; *Ucp-1*, Uncoupling protein-1.

Citrus fruits are a source of numerous phenolic compounds, pectins, and vitamins such as A, C, and E [229,230]. In recent years it has been shown that a diet rich in phenolic compounds can improve health and reduce the incidence of different diseases [231]. Some of the flavonoids found in citrus sources, such as orange, grapefruit, and lemon, are naringenin and hesperetin from the flavanones group, as well as other subtypes of flavonols, such as kaempferol, and also certain hydroxycinnamic acids [232]. Regarding obesity, one study in mice (1%) for 21 days found an increase in fatty acid oxidation in hepatocytes [233]. On the other hand, another study showed anti-inflammatory activity of naringenin in in vitro macrophages [234]. In addition, a study conducted on a murine cell line treated with *Citrus aurantium* extract (10–50 µg/mL) found inhibition of adipocyte differentiation, preventing lipid accumulation [235]. Although there are many studies that have focused on the anti-obesity effect of the phenolic compounds from citrus fruits, the effect on BAT activation and the WAT browning process has not been fully elucidated. Furthermore, a recent study demonstrated a thermogenic effect in brown adipocytes after treatment with bitter orange possible due to AMPK activation [236]. In addition, an in vivo study evaluated the effect of immature citrus fruits (1%) in HFD fed mice for 11 weeks and found an increased expression of *Ucp-1* in iWAT, thus suggesting that weight loss might be attributed to the browning process in WAT [237]. In this context, Nishikawa et al. [238] analyzed the effect of α-monoglucosyl hesperidin in mice, as a simple glycosidic flavanone, and found a significant decrease in body fat, which may be due to the activation of the browning process in iWAT. Also, another recent study evaluated the effects of naringenin on energy expenditure in human WAT (hWAT). In this context, when adipocytes were treated with naringenin for seven days, the browning process was activated through the induction of *Ucp-1* as a key marker in the thermogenesis pathway [228]. Overall, some effects of citrus flavonoids on thermogenesis have been identified, but naringenin-mediated modulation of miRNAs has not been reported to date. Figure 3 displays some of the most studied food compounds, and the main candidates target genes that promote WAT browning.

## 5. Conclusions

In summary, the detection of BAT in adult humans has raised the expectations for the development of novel anti-obesity treatments that can regulate brown or beige fat development. Recently, inducible brown adipocytes in WAT depots, called beige or brite cells, have gained more interest due to their increased capability of energy expenditure and their positive effect on diet-induced obesity. For instance, we focused on miRNAs and food compounds like key regulators of brown adipogenesis and the commitment of beige adipocytes to a brown phenotype during the “browning” process. Current knowledge deriving from clinical trials, cell culture, and animal models suggests that miRNAs can be implicated in the regulation of the critical genes involved in the differentiation and function of WAT and BAT, highlighting miRNAs as therapeutic targets for obesity. However, the dietary components discussed above have been shown to share common molecular targets involved in the induction of browning. In the long run, these findings need to be validated in more clinical trials by further large studies with a relatively long-term period of follow-up and taking into consideration factors such as ethnicity, genetics, and lifestyles.

## Figures and Tables

**Table 1:** Main miRNAs involved in brown adipocyte regulation and nutritional factors that regulate their expression in human samples/cells.

Effect on Browning	miRNA	Model and/or Tissue Sample	Nutritional Factors	Effect of the Nutritional Factor on the Expression of the miRNA	Reference
**Positive (+)**					
	203	Human colon adenocarcinoma cells (Caco-2)	Selenium (depletion/accurate levels)	↓ miR-203 expression in Se depletion	[24]
	193b	Human plasma	Weight loss dietary treatment (RESMENA trial)	↑ miR-193b levels (and hypomethylated) in high responders to weight loss	[25]
	365	Human adipocytes	Obesity/adipose (WAT) hypertrophy	↑ miR-365 in WAT hypertrophy	[26]
**Negative (−)**					
	106b	Lung cancer cells	Seed procyanidin extract (GSE) cells	↓ miR-106b in lung neoplastic cells	[27]
		LT97 cells (colon adenoma cells)	Butyrate (and trichostatin A) as histone deacetylase inhibitors	↓ miR-106b levels: affects cycle-relevant genes and thus, cell proliferation	[28]
		Plasma	Hyperlipidemia status/coronary artery disease (CAD)	↓ miR-106b in patients with CAD. MiR-106b levels positively correlate with HDL-c & ApoA-I	[29]
		Human colon adenocarcinoma cells (Caco-2)	Selenium (depletion/accurate levels)	↓ miR-106b expression in Se depletion	[24]
		HCT116 (colon cancer cells)	Butyrate (short chain FA)	↓ miR-106b expression	[30]
		Prostate cancer cells	Resveratrol treatment	↓ miR-106b expression	[31]
	93	Human hepatocellular carcinoma (HCC)	Nonalcoholic fatty liver disease (NAFLD)	↑ miR-93 expression	[32]
		Human colon adenocarcinoma cells (Caco-2)	Low-selenium environment	↓ miR-93 expression levels in low selenium	[33]
	122	Exosomes from healthy males aged 20–30 y.o.	Fat mass/BAT activity	↓ Exosomal miR-122-5p levels in high BAT activity group. Exosomal miR-12anti-obesityp correlates negatively with: BAT activity, serum HDL-cholesterol. Exosomal miR-122-5p correlates positively with: age, BMI, body fat mass, total cholesterol, and serum triglycerides.	[34]
		Plasma	Type 2 diabetes with/without NAFLD	↑ miR-122 expression in T2DM patients with NAFLD as compared to those without NAFLD	[35]
		Serum	Breast cancer patients (survivors) with different BMI ranges (obesity, weight gain)	miR-122 expression associated with BMI	[36]
		Liver samples	Patients with alcoholic liver diseases (ALD)	miR-122 levels ↓ in liver samples from ALD patients ↑ levels of miR-122 target HIF1-α	[37]
		Liver samples	Obese women following bariatric surgery with or without NAFLD	↓ miR-122 in obese subjects with NAFLD ↓ miR-122 in liver associated with impaired FA usage	[38]
		Plasma	Maternal (pre-gestational and gestational) obesity	↓ miR-122 levels in pre-gestational obesity and gestational obesity	[39]
		Human liver cancer cells (HepG2)	Flavonoid compounds (nobiletin, tangeretin, and hesperidin) from citrus peel	↓ miR-122 expression and thus, affect FAS and CPT-1α, decreasing lipid accumulation	[40]
		Serum and plasma	Individuals with risk factors for metabolic syndrome, T2D, CVD (Bruneck study)	Circulating miR-122 associated with ↑ levels of liver enzymes, adiposity, inflammation, and insulin resistance and an adverse lipid profile Circulating miR-122 levels correlated with lipid subspecies (monounsaturated and saturated fatty acids) and cholesterol esters	[41]

↑, upregulated; ↓, downregulated.

**Table 2:** Main miRNAs involved in brite adipocyte regulation and WAT browning and nutritional factors that regulate their expression in human samples/cells.

Effect on Browning	miRNA	Tissue Sample	Nutritional Factor	Effect of the Nutritional Factor on the Expression of the miRNA	Reference
**Positive (+)**					
	196a (specific)	Pancreatic cancer cells	Dietary phytochemicals (garcinol)	Modulated miR-196a expression	[42]
	26a/b	Formalin-fixed paraffin-embedded lung cancer (144 adenocarcinomas and 120 squamous cell carcinomas)	Intake of quercetin-rich foods (evaluated through a food-frequency questionnaire)	miR-26 expression differentially expressed between highest and lowest quercetin consumers	[43]
		Liver (human)	HFD/obesity	↓ miR-26 expression	[44]
	32	Healthy human colorectal epithelium	Non-digestible carbohydrates (resistant starch and polydextrose) supplementation	↑ miR-32 expression in rectal mucosa	[45]
	455	Human adipose tissue and blood cells	Obesity & T2D (computational framework miR-QTL-Scan)	BAT specific miR-455 play a role in adipogenesis	[46]
		Human adipose tissue (BAT and WAT) from neck	HFD/Obesity and cold-induced thermogenesis	MiR-455 identified as a BAT marker in humans	[47]
**Negative (** **−** **)**					
	125-5p (specific)	Blood from T2DM and obese patients	Meta-analysis including lifestyle intervention studies	↓ miR-125-5p in obese patients	[48]
	34a	Human sc WAT (48 subjects)	Three calorie-restricted diets (different amount and quality of carbohydrates): low glycemic index, high glycemic index, and low fat	↓ miR-34a in waist circumference stratified (tertiles) cohort No changes on miRNA levels between the intervention groups.	[49]
		Liver of male Sprague-Dawley rats	High-fat high-cholesterol (WD) diet supplemented with fish oil (FOH)	↓ miR-34a in FOH vs. WD	[50]
		Huh-7 (human liver) cells	Cholesterol accumulation associated to nonalcoholic fatty liver disease (NAFLD)	↑ miR-34a expression in liver	[51]
	155	THP-1 (human) monocytes/macrophages	Oleic acid	↑ miR-155 expression in monocytes (vs. DHA)	[52]
	378	Patients with NASH (liver biopsies) Human HepG2 with accumulated lipid (oleate)	N.A. Oleate	↑ miR-378 expression ↑ miR-378 expression	[53,54]
		Muscle biopsies of healthy males	Single bout of concurrent resistance exercise (8 × 5 leg extension, 80% 1RM) + 30 min at ~70% VO_2_peak with either post-exercise (whey) protein (25 g) or placebo	↑ miR-378 expression at 4 h post-exercise with protein	[55]

↑, upregulated; ↓, downregulated.

**Table 3:** Main miRNAs involved in brown adipocyte regulation and nutritional factors that regulate their expression in animal and murine cell models.

Effect on browning	miRNA	Tissue sample	Nutritional factor	Effect of the nutritional factor on the expression of the miRNA	Reference
**Positive (+)**					
	196a (specific)	Adipose tissue (sc and visceral) of lambs	DHA-G diet: barley-based finishing diet where algae meal (DHA-Gold; Schizochytrium spp.) replaced flax oil	↑ miR-196a expression in SAT vs. PAT (perirenal) in DHA-G diet. Differential miRNA expression in each tissue depot depending on diet	[56]
		Bovine adipose tissue (sc and visceral) from cattle	HFD	↓ miR-196a in HFD and higher expression in visceral tissue depot	[57]
	26a/b	Goat milk	Milk (fatty acid) composition	miR-26 expression associated with total fat yield and short-, medium and long-chain fatty acid content. No association with lactose or milk protein content. Positive correlation miR-26a family and C16:1 and C18:3 in milk fat.	[58]
		Liver and adipose tissue (pregnant) rats	Diets with different fatty acid types: soybean (SO), olive (OO), fish (FO), linseed (LO), or palm-oil (PO) diets from conception to day 12 of gestation and standard diet thereafter	MiR-26 (among others) differentially modulated by the different fatty acids during early pregnancy.	[59]
		Liver (human) & mice	HFD/obesity	↓ miR-26 expression (humans and two obesity mice models)	[44]
	30b/c	Cortex and cerebellum of middle-aged C57Bl/6J mice	Extra-virgin olive oil rich in phenols feeding for 6 months (H-EVOO, phenol dose/day: 6 mg/kg) vs. the same olive oil deprived of phenolics (L-EVOO)	↓ miR-30 expression in H-EVOO ↑ miRNAs in old L-EVOO animals compared to young.	[60]
		Rainbow trout eggs	Trout egg quality and production (characterization of miRNA profile)	miR-30 among top-10 most abundant miRNAs	[61]
		Mice adipose tissue macrophages (ATMs)	HFD (12 wk) in combination with CB1 antagonist AM251 (4 wk, 10 mg/kg)	↑ miR-30e-5p in ATMs from HFD + AM251 mice	[62]
		Male C57BL/6J mice	HFD (16 wk)	↓ miR-30a, -30c, -30e expression in ATM from HFD mice vs. NCD through epigenetic (methylation) modifications	[63]
	455	Male C57BL/6J (B6) mice	HFD (45% kcal fat) supplemented with EPA (6.75% kcal EPA) for 11 wk.	↑ miR-455 expression in BAT	[64]
		Murine adipose tissue (BAT and WAT)	HFD/Obesity and cold-induced thermogenesis	MiR-455 identified as a BAT marker in rodents	[47]
**Negative (** **−** **)**					
	27b	Milk samples from rats (lactation)	Cafeteria and post-cafeteria diet	MiR-27 levels in milk decrease throughout lactation.	[65]
		Cortex and cerebellum of middle-aged C57Bl/6J mice	Extra-virgin olive oil rich in phenols feeding for 6 months (H-EVOO, phenol dose/day: 6 mg/kg) vs. the same olive oil deprived of phenolics (L-EVOO)	↓ miR-27 levels in H-EVOO	[60]
	34a	Breast cancer cells and carcinogenesis model in rats	3,6-dihydroxyflavone (flavonoid)	↑ miR-34a in breast carcinogenesis	[66]
		Liver of male Sprague-Dawley rats	High-fat high-cholesterol (WD) diet supplemented with fish oil (FOH)	↓ miR-34a in FOH vs. WD	[50]
		Mice liver	Three dietary interventions affecting lifespan (LS): caloric restriction (CR), low fat or high fat plus voluntary exercise or 30% CR	↑ miR-34a in livers of two models of obesity MiR-34a fold change negatively correlated with LS	[67]
		Mouse pancreatic β-cells	Saturated fatty acids	↑ miR-34a expression	[68,69]
	133	C57BL/6 male mice	High fat diet concomitant with miR-133 ASO (anti-miR-133)	↑ miR-133 expression in HFD ↓ miR-133 expression in cold-exposed mice HFD and miR-133 antagonism ↑ BAT activity	[70]
	155	3T3-L1 (mouse) adipocytes	Resveratrol (25 µM)	↑ miR-155 expression	[71]
		FVB mice (colon mucosa)	High fat diet (45%) and 30% caloric restriction (CR)	↑ miR-155 expression in colon mucosa in HFD mice	[72]
		RAW264.7 macrophages (LPS activated)	10 µM quercetin, quercetin-3-glucuronide (Q3G) and isorhamnetin	↓ miR-155 expression by quercetin and Q3G	[73]
	378	Livers of dietary obese mice	HFD (60% cal.)	↑ miR-378 expression	[53,54]
		Milk samples from dairy cows in mid lactation	Control diet (total mixed ration of corn:grass silages) for 28 days followed by a treatment period (control diet supplemented with 5% linseed or safflower oil) of 28 days.	↑ miR-378 expression by both treatments	[74]
		Mice livers Mouse primary hepatocytes	Fasting & re-feed Palmitic acid (PA), linoleic acid (LA), oleic acid (OA)	↑ miR-378 expression in fasting & ↓ miR-378 upon re-feeding ↑ miR-378 expression	[29]
		Mice liver	Fisetin (a flavonoid): normal diet, HFD, HFD + fisetin	↑ miR-378 expression by HFD & ↓ miR-378 by fisetin sup.	[75]
		Mice brown adipocytes Brown fat (BAT) from mice	Omega-3 eicosapentaenoic acid (EPA) Low fat diet (LF), iso-caloric high fat (HF, 50% cal.) enriched with palm oil (HF + PO), olive oil (HF + OO), fish oil (HF + FO) for 12 wk.	↑ miR-378 expression by EPA during brown differentiation ↑ miR-378 expression in iBAT from mice treated with HF + FO vs. HF + OO or HF + PO.	[76]

↑, upregulated; ↓, downregulated.

## Abbreviations

AMPK	AMP-activated protein kinase
AMPKα1	5-AMP-activated protein kinase catalytic subunit alpha-1
BAT	Brown adipose tissue
BMP7	Bone morphogenetic protein 7
BW	Body weight
C/EBPα	CCAAT/enhancer binding protein alpha
Cd137	4-1BB
Cd40	CD40 molecule
CIDEA	Cell death inducing DFFA like effector A
CITED1	Cpb/P300 interacting transactivator with Glu/Asp rich carboxy-terminal
CLA	Conjugated linoleic acid
Cox8	Cytochrome c oxidase subunit 8
CPTB1β	Carnitine palmitoyltransferase I
DHA	Docosahexanoic acid
Dio2	Iodothyronine deiodinase 2
EGCG	Epigallocatechin gallate
Elovl3	Elongation of very long-chain fatty acid 3
EPA	Eicosapentanoic acid
ERK	Extracellular signal-regulated kinases
eWAT	Epididymal WAT
Fgf32	Fibroblast growth factor 23
FNDC5	Fibronectin type III domain-containing protein 5
Foxc2	Forkhead box C2
HFD	High-fat diet
HUVECs	Human umbilical vein endothelial cells
hWAT	Human white adipose tissue
iWAT	Inguinal WAT
LA	Linoleic acid
LFD	Low-fat diet
LPS	Lipopolysaccharides
MAPK	Mitogen-activated protein kinase
miRNAs	MicroRNAs
mWAT	Mesenteric WAT
Myf5	Myogenic factor 5
NAFLD	Nonalcoholic fatty liver disease
OPE	Onion peel extracti
Pgc-1α	Peroxisome proliferator-activated γ receptor co-activator 1 alpha
Ppargc1b	PPARG coactivator 1 beta
Pparα	Peroxisome proliferator-activated receptor α
Pparγ	Peroxisome proliferator-activated receptor gamma
Pparδ	Peroxisome proliferator-activated receptor delta
Prmd16	PR-domain containing 16
PUFA	Polyunsaturated fatty acids
RA	Retinoic acid
RAR	Retinoic acid receptor
RNA	Ribonucleic acid
rWAT	Retroperitoneal WAT
RXR	Retinoid X receptor
Sirt-1	Sirtuin 1
sWAT	Subcutaneous WAT
Tbx1	T-box 1
tCA	Trans-cinnamic acid
TG	Triglycerides
Tmem26	Transmembrane protein 26
TRPM8	Transient receptor potential cation channel subfamily M (melastatin) member 8
TRPV1	Transient receptor potential vanilloid subfamily 1
UCP-1	Uncoupling protein-1
UCP-2	Uncoupling protein-2
VEGFα	Vascular endothelial growth factor alpha
WAT	White adipose tissue
β3-AR	Beta3 adrenergic receptor
